# Circulating Tumor Cell Count Correlates with Colorectal Neoplasm Progression and Is a Prognostic Marker for Distant Metastasis in Non-Metastatic Patients

**DOI:** 10.1038/srep24517

**Published:** 2016-04-14

**Authors:** Wen-Sy Tsai, Jinn-Shiun Chen, Hung-Jen Shao, Jen-Chia Wu, Jr-Ming Lai, Si-Hong Lu, Tsung-Fu Hung, Yen-Chi Chiu, Jeng-Fu You, Pao-Shiu Hsieh, Chien-Yuh Yeh, Hsin-Yuan Hung, Sum-Fu Chiang, Geng-Ping Lin, Reiping Tang, Ying-Chih Chang

**Affiliations:** 1 Division of Colon and Rectal Surgery, Colorectal Section, Department of Surgery, Chang Gung Memorial Hospital, School of Medicine, Chang Gung University, 5 Fu-Hsing St. Kueishan, Taoyuan, Taiwan; 2 Graduate Institute of Clinical Medical Science, Chang Gung University, Taoyuan, Taiwan; 3 Genomics Research Center, Academia Sinica, 128, Sec. 2, Academic Rd., Nankang, Taipei 115, Taiwan; 4 Graduate Institute of Life Sciences, National Defense Medical Center, Taipei 114, Taiwan

## Abstract

Enumeration of circulating tumor cells (CTCs) has been proven as a prognostic marker for metastatic colorectal cancer (m-CRC) patients. However, the currently available techniques for capturing and enumerating CTCs lack of required sensitivity to be applicable as a prognostic marker for non-metastatic patients as CTCs are even more rare. We have developed a microfluidic device utilizing antibody-conjugated non-fouling coating to eliminate nonspecific binding and to promote the multivalent binding of target cells. We then established the correlation of CTC counts and neoplasm progression through applying this platform to capture and enumerate CTCs in 2 mL of peripheral blood from healthy (n = 27), benign (n = 21), non-metastatic (n = 95), and m-CRC (n = 15) patients. The results showed that the CTC counts progressed from 0, 1, 5, to 36. Importantly, after 2-year follow-up on the non-metastatic CRC patients, we found that those who had ≥5 CTCs were 8 times more likely to develop distant metastasis within one year after curable surgery than those who had <5. In conclusion, by employing a sensitive device, CTC counts show good correlation with colorectal neoplasm, thus CTC may be as a simple, independent prognostic marker for the non-metastatic CRC patients who are at high risk of early recurrence.

Colorectal cancer (CRC) is among the most common cancers in Taiwan and worldwide^1^. Clinically, despite having received radical surgery and adjuvant treatment, about 25–50% of patients with Stage II or III CRC suffer from tumor relapse as a consequence of initially undetectable metastasis^2^. The prognosis of patients whose CRC has spread to distant sites is often poor^3^. Clinicopathologic factors such as perforation or obstruction of the bowel by the tumor, depth of tumor invasion, regional lymph node metastasis, vascular invasion, and cell shape differentiation, significantly influence the prognosis in early stages of CRC; however, these parameters remain inaccurate in their ability to establish the prognosis of individual non-metastatic CRC patients. In order to identify such patients who are at high risk of developing metastases and thus may benefit from early treatment, there is a strong need for a technique that can detect colorectal tumor cell spread in its earliest stages, before distant metastatic lesions have formed, and with high sensitivity.

The first step in the metastatic process is the detachment of tumor cells from the primary tumor. When a tumor grows beyond 2 mm^3^, it can undergo angiogenesis^4^, or new blood vessel formation, which allows tumor cells that have detached from the primary tumor to enter the vascular system and potentially circulate to distant locations, such as the lungs or the liver. Tumor cells present in the peripheral bloodstream, or circulating tumor cells (CTCs), are rare, existing at 1 CTC per 10^6^–10^9^ blood cells, or even lower when solid tumors are confined to local growth. Based on experimental *in vivo* studies, CTCs are thought to be present early in the natural history of solid tumor growth, before the development of metastasis^5^.

The most widely used commercial platform for enumerating CTCs, the Veridex CellSearch system, which has US FDA 510(k) clearance, involves utilizing the antibody of the epithelial cell adhesion molecule (anti-EpCAM) coated on magnetic beads to capture cells and then immunostaining those cells to identify CTCs, defined in the CellSearch system as cytokeratin (CK)8/18/19^+^/DAPI^+^/CD45^−^ cells. However, the CTC detection rates and counts in the CellSearch system generally are low. For example, for the metastatic CRC patients (n = 413), the median CTC counts per 7.5 mL peripheral blood was 0^6^. For the non-metastatic CRC patients group, in one study, ≥2 CTCs per 7.5 mL of peripheral blood were present in 26% of 66 non-metastatic CRC patients^7^, and in another study, only in 5% (1 in 20 preoperative non-metastatic CRC patients). Recent study showed that ~8% (19 out of 239 preoperative non-metastatic CRC patients) had ≥1 CTC per 7.5 mL blood with median value of 0. However, even with the low abundance of CTC, the study indicated that CTC is the strongest prognostic factor in non-metastatic patients^8^. This led to the conclusion that a more sensitive CTC detection assay can further facilitate the evaluation of CTC levels as an independent prognostic marker, particularly in non-metastatic CRC patients^9^.

Here we show a sensitive CTC capture platform (“CMx” platform- **C**TCs in **M**a**x**imum) composed of a coating of anti-EpCAM-functionalized supported lipid bilayer (SLB), a non-fouling lubricant-like membrane, in a chaotic mixing microfluidic chip^10^,^11^,^12^. The anti-EpCAM functionalized SLB not only allows for high-efficiency capture of CTCs but, equally importantly, can be easily disintegrated using air foam, hence eluting viable CTCs from the inside of channels. The released CTCs can then be concentrated on a 1-cm^2^ planar, porous substrate for convenient immunofluorescent staining and imaging.

We previously showed that the CMx platform was successfully used to capture 97% of CRC cells from HCT116 cancer line that had been spiked into whole blood. The cancer cells were subsequently eluted with an overall purity of greater than 95%^10^. Additionally, our recent study also demonstrated CK20/CDX2^+^ CTCs in 3 of 5 early stage CRC blood samples^13^. In the present study, we utilized the CMx platform to detect CTCs, which we define as CK20^+^/DAPI^+^/CD45^−^ cells, in 2 mL of peripheral blood from individuals with normal colon, benign colon disease, and preoperative non-metastatic and metastatic CRC. Our main goal was to use the CMx platform to identify correlations between CTC count and both colorectal neoplasm progression and the appearance of distant metastases in non-metastatic CRC patients. Insights gleaned from this research would allow us to determine whether CTCs might be useful for identifying individuals who are at high risk of developing metastatic CRC and hence may benefit from early treatment.

## Results

### Distribution of CTC counts and detection rates in each classification

**Figure 1:**
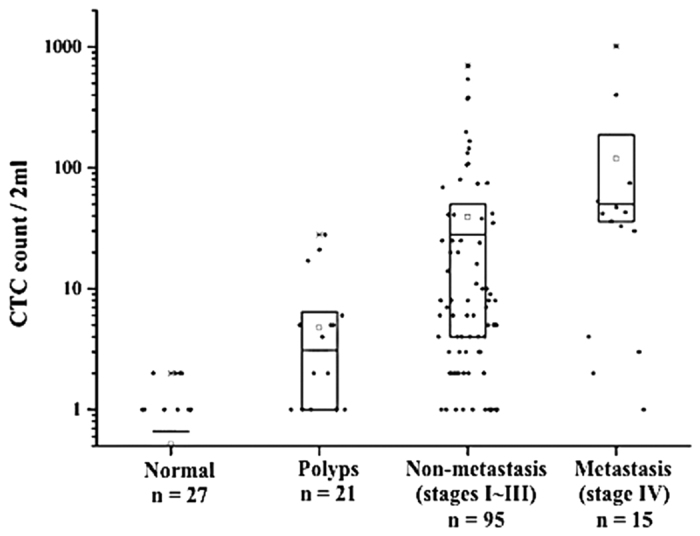
CTC count in different patient groups. The square in the box represents the mean. The upper and middle line of the box represent the mean ± 1 σ. The bottom line of the box represents the median.

The CTC counts for healthy patients, patients with polyps, and CRC patients are presented in Table 1 and Fig. 1. The mean value of CTCs in the group of patients diagnosed with CRC was significantly higher than that of the healthy group (P < 0.001) and the group with polyps (P = 0.001). Within each subgroup, the mean and median CTC numbers increased with the severity of the subgroup’s condition. For example, in the group with colorectal polyps, the mean count of CRCs increased from 1 to 5 to 8 for the subgroups with hyperplastic, adenomatous, and severely dysplastic polyps, respectively, while the mean count of CRCs in the healthy group was 0. Similarly, in the cancer group, the mean CTC values were 39 and 119 for the non-metastatic and metastatic CRC patients, respectively. Linear regression of the mean values of CTC counts from healthy patients, patients with benign polyps, and patients with CRC yielded an R^2^ value of 0.071, with P = 0.001. Together, these results reveal that CTCs are present at an early stage of neoplasm formation and that CTC count correlates well with neoplasm progression.

**Figure 2:**
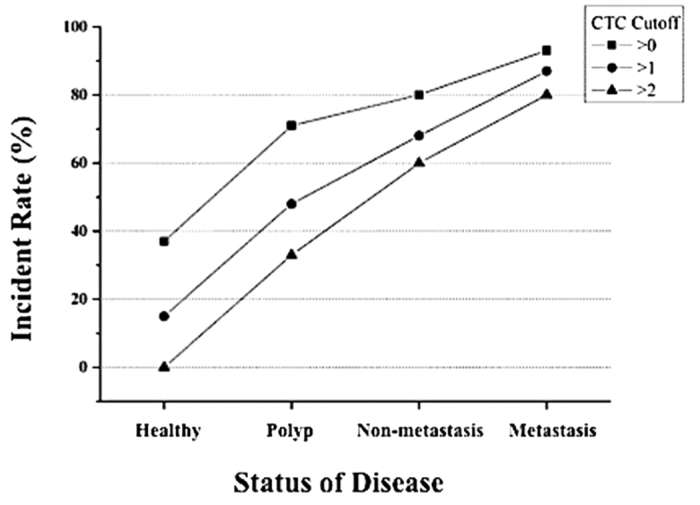
Percentage of patients that had CTC counts above the shown cutoff values as a function of disease status. Three levels of CTC count cutoffs were used: >0, >1, or >2, all per 2 mL of blood sample.

We used the data presented in Table 1 to determine the CTC count cutoff that would be indicative of CRC. Figure 2 shows the percent of patients of each group that had a CTC count higher than each of the cutoff values used in the graph (CTC > 0, 1, or 2 per 2 mL). For each cutoff value, the positive incidence rates increased with disease severity. Using >2 CTCs as a cutoff, 0% of the healthy patients had CTC counts greater than the cutoff. The percentage of patients with CTC counts >2 were 33%, 62%, and 80% for patients with polyps, non-metastatic CRC, and metastatic CRC, respectively (P < 0.001). Because of these results, we used a CTC cutoff of >2 per 2 mL of blood in the rest of our analyses, except where noted.

**Figure 3:**
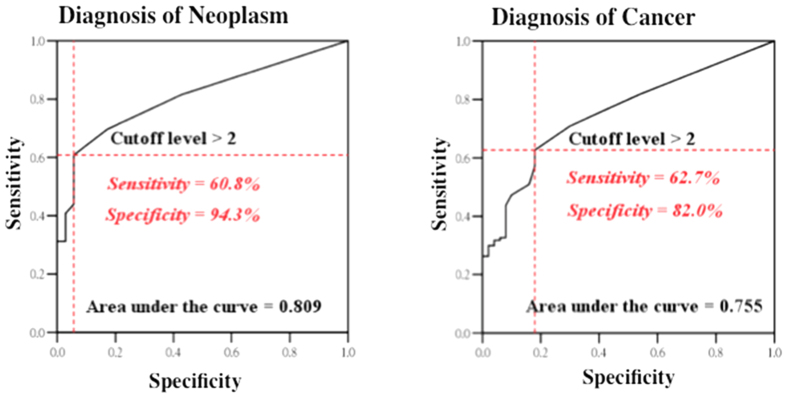
Receiver operating characteristic curve of diagnosis of neoplasm presence (normal + hyplastic polyp vs. adenomatous/dysplasic polyp + CRC) and CRC (normal + polyps vs. CRC) using a CTC count of >2 cells per 2 mL peripheral blood as the diagnostic indicator.

Based on this cutoff, the sensitivity and specificity were 63% and 82%, respectively, for the diagnosis of cancer (receiver operating characteristic (ROC) curve, area under curve (AUC) = 0.755), and 61% and 94%, respectively, for the diagnosis of colorectal neoplasm, including adenomatous polyps, dysplastic polyps, and cancer (ROC curve, AUC = 0.809, Fig. 3). The positive detection rates were 3, 47, 35, 66, 67, and 80.0% in the groups of normal/hyperplastic polyp, adenomatous/dysplastic polyp, stages 0/I, II, III, and IV, respectively. The high specificity for diagnosis of neoplasm in the ROC curve and the low positive detection rate in the healthy/hyperplastic group indicate that CTCs may be a potential marker for diagnosis of colon neoplasm.

### Relationships between CTC number and clinicopathologic characteristics in non-metastatic CRC cases

Ninety-five non-metastatic CRC cases were involved in this analysis. The distributions of CTC counts for patients grouped by various clinicopathologic characteristics are shown in Table 2. The mean CTC number increased gradually with advancement of tumor and nodule (T, N) classification, tumor size, and histological differentiation (all P <0.05). There was no correlation between CTC count and sex, age, CEA level, tumor location, or gross type. The CTC number of patients with bowel obstruction caused by tumor was statistically higher than that of patients without obstruction (P < 0.001). These results show that the presence of CTCs correlated well with tumor progression, which is consistent with the results presented in Table 1 and Fig. 1, and suggest that intravasation of tumor cells is promoted by bowel obstruction.

### High CTC count related to distant metastasis

**Figure 4:**
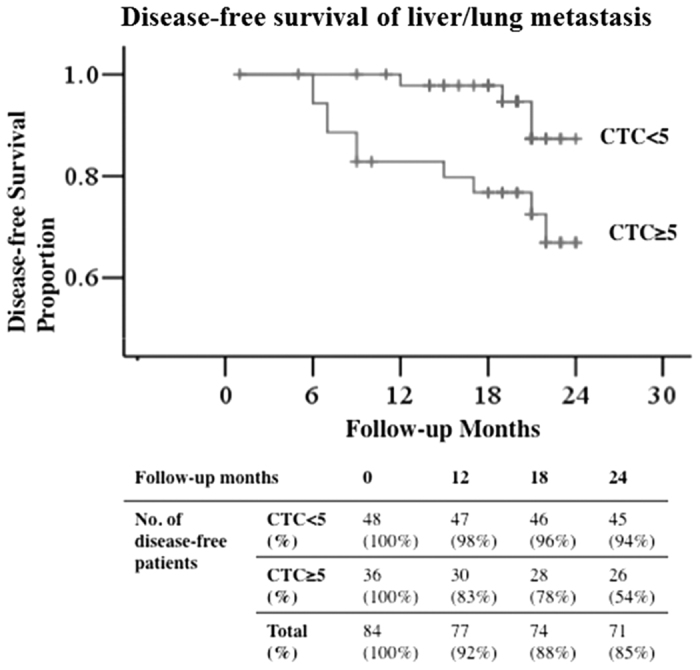
A comparison of the relationship between CTC count and the disease-free survival (DFS) proportion over time, of distant (liver or lung) metastasis. Those patients with an initial CTC count of >5 cells per 2 mL of blood had a statistically significantly greater likelihood of developing a distant metastasis.

A comparison of the non-metastatic CRC patients showed that those with a high CTC count (≥5 cells per 2 mL blood sample) were more likely to develop distant metastasis than those with a CTC count <5. Excluding 9 patients who had a tumor obstruction, the 2-year disease-free survival from distant metastasis, referring to liver or lung metastasis in this work, of the total 84 CRC patients was analyzed. Nineteen cases (1 stage I, 4 stage II and 14 stage III) suffered from either distant metastasis (6 cases of liver, 5 cases of lung, 2 cases of liver and lung) or peritoneal/local metastasis (6 cases). As shown in Fig. 4, there was a statistically significant difference (Kaplan-Meier method, P = 0.015) in the 2-year disease-free survival rate from distant metastasis for patients with CTC counts <5 versus those with CTC counts ≥5 cells, with disease-free survival rates of 91% and 71%, respectively. However, there was no significant distinction of CTC counts for the occurrence of local/peritoneal metastasis.

Multivariate analysis of the disease-free survival data of our patient group showed that a CTC count ≥5 was an independent prognostic factor of distant metastasis (Hazard ratio = 7.5, 95% CI: 1.6 to 34.7, P = 0.01). N classification, tumor size, and tumor location were also independent prognostic factors, as expected. In contrast, the factors of CEA level, T classification, and histological differentiation were not prognostic factors (Table 3). The mean preoperative CTC number in patients who later had a distant metastasis was 51 ± 147 cells (median = 8), which was significantly higher than that of patients who had no metastasis, 19 ± 53 cells (median = 3, P = 0.01).

Differences in the 1-year survival rate from distant metastasis were even more apparent than differences in the 2-year survival rate. In 12 months, 17% of the patients who initially had CTC counts ≥5 were diagnosed with a distant metastasis. In contrast, in 12 months only 2% of the patients with an initial CTC count <5 were diagnosed with a distant metastasis (Fig. 4).

## Discussion

CTC detection from peripheral blood has the advantage in that it can be performed easily, frequently, and less invasively than most diagnostic methods. There is growing evidence to support CTCs as a clinical marker for diagnostic, prognostic, and pharmacologic purposes^14^. Although various CTC isolation techniques have been developed recently, all of the CTC studies that proved that high baseline CTC counts were associated with worse prognosis in metastatic breast^15^, prostate^14^, and colon cancer^6^ employed the CellSearch system. However, clinical studies using CellSearch have shown that this technique is not suitable for the detection of early tumor relapse in non-metastatic CRC patients. For example, the percentages of patients with >1 CTC per 7.5 ml blood using CellSearch were 21% in stage II (n = 29) and 24% in stage III (n = 29)^7^. Another study reported only a 5% positive CTC detection rate for 20 non-metastatic CRC patients^9^. In comparison, our use of the CMx platform and a cutoff of >2 CTCs per 2 mL to analyze the non-metastatic CRC patients in this study yielded positive CTC detection rates of 35% in stage 0/I (n = 20), 66% in stage II (n = 29), and 67% in stage III (n = 46), significantly higher than those detected by CellSearch system. The high sensitivity of CMx platform could be mainly attributed to two factors: First, CMx platform captures cells using a non-fouling, lubricated coating in the microfluidic chip^10^, thus reducing the shear stress and allowing for efficient cell purification and detachment while preserving cell viability and morphology^11^. Secondly, we used CK20 as a sensitive and specific staining marker, which is more sensitive for colon originated CTCs, comparing to the conventional CK8/18/19^16^,^17^.

We observed that certain clinical conditions could influence CTC counts, as shown in Table 2. Higher CTC counts were detected in patients with bowel obstruction, indicating that bowel wall distention due to obstruction and mucosal breaking due to perforation promote epithelial cells shedding into circulation. This is consistent with previous clinical findings that bowel obstruction is a poor prognostic factor for CRC patients^18^. According to the results of a large-scale study including 2,068 CRC cases, the 5-year cancer-specific survival rates following curative surgery were 61%, 52%, and 46% of patients with blood loss, obstruction, and perforation, respectively. These values are statistically significantly lower than the rate of 75% of those patients without these clinical symptoms. The corresponding adjusted hazard ratios (95% confidence interval) for cancer-specific survival, relative to elective patients, were 1.62 (1.22 to 2.15), 2.22 (1.78 to 2.75), and 2.93 (1.82 to 4·70), respectively (all P < 0.001)^19^.

Higher CTC counts were also detected in patients with previous polypectomy (data not shown). An animal model study showed that electrocautery snare resection stimulates cellular proliferation of residual colorectal tumor^20^. This may explain the higher CTC counts in the patients with previous polypectomy in our study.

Using the CMx platform, we detected CTCs in patients with benign polyps, which is consistent with the results of a CellSearch study^21^. However, our detection rate was much higher: 38% of patients with polyps were CTC positive (i.e., had >2 CTCs per 2 mL) in our study, versus 1 out of 12 (8.3%) CTC positive (had >0 CTC per 7.5 mL) in the CellSearch study^21^. Furthermore, using the CMx platform a positive trend between CTC count (or detection rates) and the progression from hyperplastic to adenomatous and dysplastic polyps could be readily established (Table 1). To our knowledge, we are the first to establish such a trend.

It is important to note that not all CTCs develop into CRC, due to spontaneous apoptosis in circulation, intervention such as adjuvant chemotherapy, or the inability of certain subpopulations of CTCs to form tumors at secondary sites. In order to help identify more specific subpopulations of CTCs with metastasis potential, the next challenge will be to serially examine CTC count from preoperative to postoperative periods and perform molecular analysis of CTCs. Nevertheless, the presence of CTCs in presumably benign polyps strongly suggests that CTCs could be viewed as an early warning of cancers with a more aggressive tendency to extravasate and a greater potential for metastasis.

In our study, we showed that higher preoperative CTCs were associated with a high risk of distant metastasis. This result reveals that preoperative CTC detection can be used as a biomarker to select the patients with high risk of hematogenous spreading metastasis for preventive adjuvant chemotherapy. The presence of postoperative CTCs is likely to indicate occult metastasis because a recent report showed that 75% of 69 CRC patients undergoing radical surgery with high postoperative levels of CTCs detected in peripheral bloods experienced tumor recurrence^22^. Thus, postoperative CTC enumeration can be used as an early indicator of otherwise undetectable metastasis, thereby improving the odds of the patient’s survival.

In conclusion, our data show that both CTC detection rates and CTC counts have positive correlation with neoplasm progression and the appearance of distant metastases in non-metastatic CRC patients. We have developed a strategy to retrieve and identify CTCs with high sensitivity and specificity, and we used this strategy to demonstrate statistically significant correlation between preoperative CTC count and colorectal neoplasm progression from early to advanced stages. Using a small volume of blood (2 mL), we have identified a sizable portion of polyp patients and non-metastatic CRC patients with >2 CTCs. This cutoff could potentially be used clinically to discern patients with colorectal disease from healthy individuals. The follow-up on the CRC patients with high CTC counts showed that they are subject to high risk of early development of distant metastasis, even after a curable tumor resection. This study has demonstrated that CTCs may be useful clinically for diagnosing colorectal neoplasms and for identifying non-metastatic CRC patients who are at high risk of developing distant metastases and hence may benefit from early treatment.

## Methods

### Patients

From July 4, 2012 to November 1, 2012, 158 patients prior to colonoscopy examination or suspected of having CRC with unconfirmed clinical stages, were recruited at Chang Gung Memorial Hospital, Linkou Campus. The blood samples of cancer patients were obtained before curative resection of tumor in patients with non-metastatic CRC and before chemotherapy in patients with metastatic CRC. This prospective study was double-blinded in terms of both blood draw and CTC enumeration.

The TNM classification of the tumor was based on AJCC 7^th^ edition. All of the clinicopathologic parameters of patients were classified according to the chart records. Donors with neoadjuvant therapy, other known cancers, and multiple GI tract diseases (such as polyps and ulcerative colitis) were excluded from the analysis.

Based on colonoscopic exams and chart records, the volunteers consisted of 110 new CRC patients (15 stage IV, 95 stages 0-III), 21 patients with benign polyps, and 27 healthy patients. For the purpose of this study, “healthy” means that the patient had no previously known cancer and did not have CRC or polyps at the time of the study.

The ages of the people in the reference group, which consisted of the patients with polyps and the healthy patients, ranged from 25 to 76 years old, with a mean age of 51 ± 14 years old. The reference group was significantly younger (P < 0.001) than the cancer group, whose ages ranged from 34 to 87 years old, with a mean of 62 ± 12 years old.

Except for one case who did not undergo surgical resection, all other non-metastatic cases (n = 94) had undergone curative or intended curative resection of the tumor within one day after their blood was drawn for CTC enumeration. One patient was suffered from post-operative mortality. With the exception of these two, total 93 non-metastatic CRC patients regularly received follow-up for at least 2 years until November 2014, with a mean of 25.6 months if no recurrence. The clinical exams included serum CEA measurements every 3 months, and abdominal sonography or CT scan, chest X-ray, and colonoscopy every 12 months or at the time of suspected metastasis by clinical symptoms or CEA level. Informed consent with IRB approval (No.100–4274B of Ethic Committee of Chang Gung Memorial Hospital, and AS-IRB01-11056 in Academia Sinica) had been obtained from all cases before examination. Blood samples were collected in accordance with the approved guidelines by the Chang Gung Memorial Hospital, all experiments were performed following protocols as approved by the ethics committee.

### CMx platform CTC enumeration

The CMx platform was used for CTC isolation and enrichment^10^,^11^. The procedure of this method is as follows. First, peripheral blood samples from donors were drawn and collected into Vacutainer tubes containing the anticoagulant EDTA (BD Biosciences). Then 2 mL of blood was infused into the anti-EpCAM-SLB coated chip at a flow-rate of 1.5 mL/h. After the completion of the blood infusion, phosphate-buffered saline was used to wash out the loosely bound cells. Subsequently, air foam was infused into the chip to disintegrate the SLB, thus eluting the captured cells. The released cells were stained with DAPI, anti-CK20 antibody, and anti-CD45 antibody. Round particles with cell morphology that were 7–25 μm in diameter and CK20 + in the cytoplasm, DAPI + in the nucleus, and CD45- were defined as CTCs and counted under microscopy with a Leica DM-IRE2 (Automatic Inverted Microscope and Living Cell System) and Leica AF 6000 (Advanced Fluorescence Imaging System) (Supplementary Figure S1).

The ability of the CMx system to enumerate accurately the CTCs was verified with a daily control study. The CRC cell line HCT116 was spiked into a 2-mL blood sample from a healthy donor, which was simultaneously processed and used as a control. In a study performed in triplicate, 75% of HCT116 cells (5~1000 cells in 2 mL blood) were captured consistently (Supplementary Figure S2). HCT116 cell line was purchased from Bioresource Collection and Research Center (BCRC, Taiwan) and used within 6 months. The STR-PCR profile characterization of cell lines was done routinely by BCRC. At least two trained operators independently assessed each cell image, and the CTCs were counted based on consensus.

### Statistical analysis

Statistical analysis was performed using SPSS for Windows (Version. 12.0, SPSS Inc, Chicago, IL). Two-independent-samples T test and ANOVA analyses were used to compare the mean value and linearity between different groups. Linear regression test was used to test the linear association between groups. Pearson’s chi-square test was used to analyze the differences in the incidence of positive CTC detection rates between groups. Survival analysis was performed by the Kaplan-Meier method and a log-rank test. The Cox regression model was used for multivariate analysis of prognosis factors. All P-values were two-sided. P-values of less than 0.05 indicated statistical significance.

## Additional Information

**How to cite this article**: Tsai, W.-S. *et al.* Circulating Tumor Cell Count Correlates with Colorectal Neoplasm Progression and Is a Prognostic Marker for Distant Metastasis in Non-Metastatic Patients. *Sci. Rep.* **6**, 24517; doi: 10.1038/srep24517 (2016).

## Supplementary Material

Supplementary Information

## Figures and Tables

**Table 1:** Number of CTCs detected in reference and cancer cases.

	No. of cases	CTC count	
		Mean ± SD	Range (Median)
*Reference cases*	48	2.6 ± 5.6*	0–28 (1)
Normal colon	27	0.5 ± 0.8	0–2 (0)
Polyp	21		
Hyperplastic polyp	6	1.3 ± 2.3	0–6 (0.5)
Adenomatous polyp	11	5.3 ± 7.1	0–21 (2)
Dysplastic polyp	4	8.5 ± 13.1	1–28 (2.5)
*Colorectal cancer cases*	110	50 ±140.6*	0–1012 (5)
Non-metastatic CRC	95	39 ± 107.3	0–699 (4)
Stage 0/I	20	7.3 ± 23.8	0–108 (1)
Stage II	29	31.4 ± 73.7	0–381 (4)
Stage III	46	57.9 ± 140	0–699 (6.5)
Metastatic CRC (Stage IV)	15	118.7 ± 266**	0–1012 (36)

*P < 0.05 when compared to each other.

**P < 0.05 when compared the mean value for non-metastatic CRC patients.

**Table 2:** Distribution of circulating tumor cell counts of different groupings of non-metastatic cancer patients.

	No. of cases	No. of CTCs
Age		
≤70	70	38.8 ± 107.2
>70	25	39.9 ± 109.8
Sex		
Male	51	35.1 ± 84.3
Female	44	43.8 ± 129.8
Tumor location		
Colon	60	42.8 ± 114.3
Rectum	35	32.9 ± 95.4
Bowel obstruction by tumor		
No	86	24.3 ± 74.7
Yes	9	181.1 ± 228.7*
CEA level (μg/L)		
≤5	70	35.0 ± 99.7
>5	25	50.5 ± 127.8
Tumor size		
<2 cm	15	12.3 ± 28.5
2 ~ 5 cm	54	17.5 ± 29.1
>5 cm	25	97.7 ± 192.9^*^
Gross type		
Polypoid	17	29.2 ± 52.3
Ulcerative	74	38.7 ± 116.9
Infiltrative	3	69.3 ± 111.4
T classification		
Tis	4	4 ± 3.7
T1	8	19.5 ± 38.4
T2	12	10.4 ± 30.2
T3	60	29.9 ± 85.8
T4	10	148.3 ± 229.5^*^
N class		
N0	49	21.5 ± 59.5
N1	23	25.9 ± 76.4
N2	22	87.3 ± 182.9^*^
Histologic differentiation		
Well	5	12.0 ± 17.0
Moderate	76	30.5 ± 94.0
Poor	13	91.6 ± 173.6^*^

*P < 0.05 when compared to corresponding groups of the same classification.

CEA: carcinoembryonic antigen.

**Table 3:** Cox Regression multvariate analysis of prognostic factors for liver/lung metastasis.

	P value	Hazard ratio	95% Confidence interval
CTC count, ≥5 vs. <5	0.01	7.5	1.6 to 34.7
T classification, 1 to 3 vs. 4	0.456	2	0.3 to 13.4
N calssification, negative vs. positive	0.029	6.4	1.2 to 33.7
Tumor size, ≤5 vs. >5cm	0.472	0.6	0.1 to 2.6
Histology differentiation, well/moderate vs. poor	0.521	1.8	0.3 to 10.7
Tumor location, colon vs. rectum	0.136	2.6	0.7 to 9.1
CEA elevation, ≤5 vs. >5 ng/ml	0.04	4.7	1.1 to 20.3
