# Supplementary material for: Circulating Tumor Cell Count Correlates with Colorectal Neoplasm Progression and Is a Prognostic Marker for Distant Metastasis in Non-Metastatic Patients
Source: Sci Rep. 2016 Apr 14;6:24517. doi: 10.1038/srep24517 (PMC4830949; doi:10.1038/srep24517)
Supplement: Supplementary Information [file srep24517-s1.pdf]

Supplementary Information for

Circulating Tumor Cell Count Correlates with Colorectal Neoplasm Progression and Is a Prognostic Marker for Distant Metastasis in Non-Metastatic Patients

Wen-Sy Tsai[a, b], Jinn-Shiun Chen[a, b], Hung-Jen Shao[c, e], Jen-Chia Wu[c, e], Jr-Ming Lai[c, e], Si-Hong Lu[c, d], Tsung-Fu Hung[c], Yen-Chi Chiu[c, e], Jeng-Fu You[a], Pao-Shiu Hsieh[a], Chien-Yuh Yeh[a], Hsin-Yuan Hung[a], Sum-Fu Chiang[a], Geng-Ping Lin[a], Reiping Tang[a], Ying-Chih Chang[c, d, *]

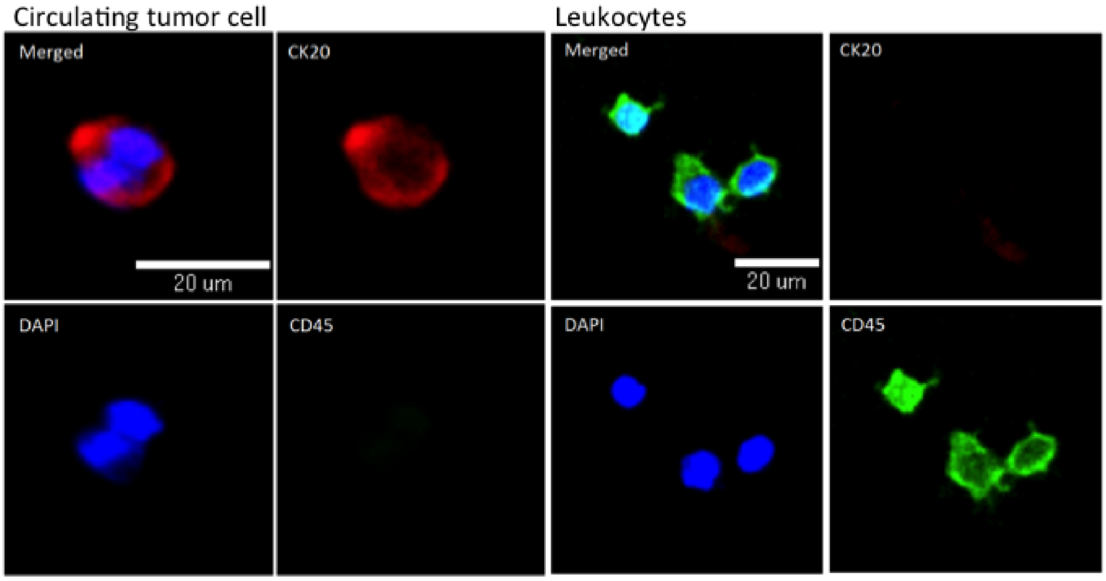

Supplementary Figure S1. Images of CTCs and leukocytes taken from blood samples of colorectal cancer patients. The tumor cells were stained with anti-CK20 antibody, DAPI, and anti-CD45 antibody.

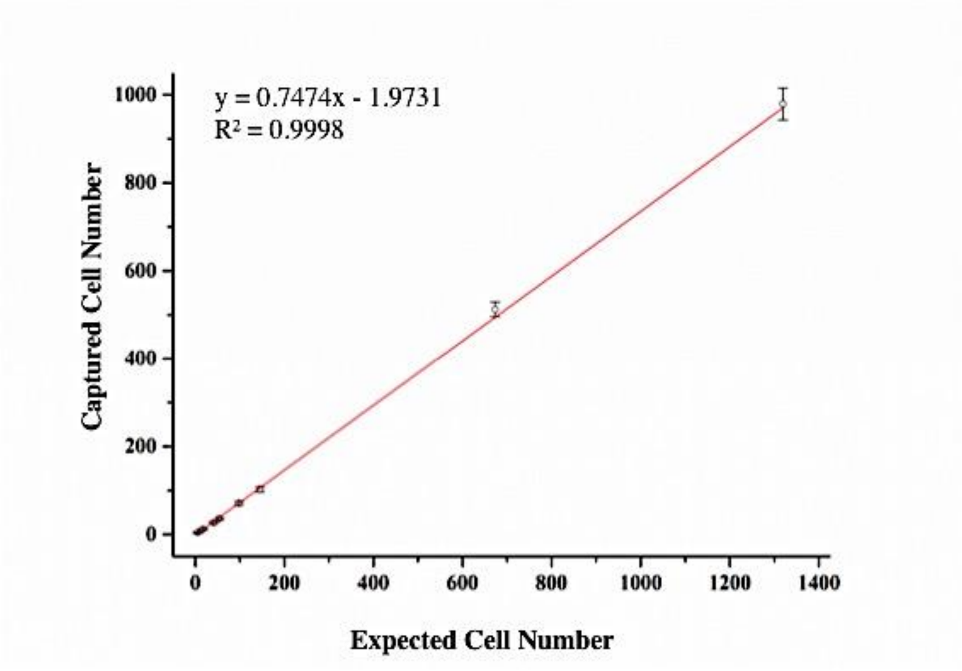

Supplementary Figure S2. Comparison of the expected CTC count to the captured CTC cell count in a control study of the CMx cell counting process using the HCT116 colon cancer cell line. The linear fit shows that 75% of the cancer cells were captured over the range of cell counts used (5–1000 cells).
